# Improving Health Care Management in Primary Care for Homeless People: A Literature Review

**DOI:** 10.3390/ijerph15020309

**Published:** 2018-02-10

**Authors:** Maeva Jego, Julien Abcaya, Diana-Elena Ștefan, Céline Calvet-Montredon, Stéphanie Gentile

**Affiliations:** 1 EA 3279 Research Unit—Public Health, Chronic Diseases and Quality of Life, Faculty of Medicine, Aix-Marseille University, 27 Bd Jean Moulin, 13385 Marseille CEDEX 5, France; StephanieMarie.GENTILE@ap-hm.fr; 2 Department of General Practice, Faculty of Medicine, Aix-Marseille University, 27 Bd Jean Moulin, 13385 Marseille CEDEX 5, France; abcaya.julien@gmail.com (J.A.); celine.montredon@hotmail.fr (C.C.-M.); 3 Faculty of Medicine, Carol Davila University of Medicine and Pharmacy, 37 Street Dionisie Lupu, Sector 1, 030167 Bucharest, Romania; anaidpopescu@gmail.com

**Keywords:** homeless persons, primary health care, access to health care, health services accessibility

## Abstract

Background: Homeless people have poorer health status than the general population. They need complex care management, because of associated medical troubles (somatic and psychiatric) and social difficulties. We aimed to describe the main characteristics of the primary care programs that take care of homeless people, and to identify which could be most relevant. Methods: We performed a literature review that included articles which described and evaluated primary care programs for homeless people. Results: Most of the programs presented a team-based approach, multidisciplinary and/or integrated care. They often proposed co-located services between somatic health services, mental health services and social support services. They also tried to answer to the specific needs of homeless people. Some characteristics of these programs were associated with significant positive outcomes: tailored primary care organizations, clinic orientation, multidisciplinary team-based models which included primary care physicians and clinic nurses, integration of social support, and engagement in the community’s health. Conclusions: Primary health care programs that aimed at taking care of the homeless people should emphasize a multidisciplinary approach and should consider an integrated (mental, somatic and social) care model.

## 1. Introduction

Homelessness has increased during the past 30 years in high-income countries [1]. It is estimated that 4 million people experience homelessness each year in the European Union, and more than 2.5 million in the USA [1]. The European Typology on Homelessness and Housing Exclusion (ETHOS) offers a harmonized definition of homelessness. It considers four conditions: rooflessness (living rough or in emergency shelters), houselessness (people living in shelters, more long-term accommodations or due to being released from institutions), insecure housing, and inadequate housing (for example living in caravans on illegal campsites, living in unfit housing, or extreme overcrowding) [2]. In this definition homelessness reflects precariousness as a dynamic process where the accumulation of health and/or social factors of vulnerability lead at the end to social exclusion. In France, the Foundation Abbé Pierre’s 2017 annual report estimated that about 900,000 persons lacked personal housing (living rough, in shelters, in hostels, with their family or their friends, or in inadequate/non-conventional accommodations), and about 4 million were homeless or experienced hard housing conditions [3]. More and more youth, women and families are affected by homelessness in Europe [1,4]. The homeless population is also ageing with a median age of 50 years in the USA [1]. Homeless people need complex health care, including medical (somatic and mental health care), psychological, and social support. Indeed, they have worse physical and mental health status and suffer from higher mortality than housed people [1,5,6]. Their poor health status is marked by chronic conditions, mental health problems, and substance use problems [7,8,9]. Prevalence rates for mental disorders go from 30% to more than 60%, and more than 50% of homeless people have concurrent substance addiction and mental disorders [9,10,11]. However, they face multiple difficulties in accessing primary health care and receive less preventive health care than the general population [12,13]. The encountered barriers can be: the lack of proof of health insurance, difficulties in keeping appointments, a bad experience of care and the fear of discrimination, or competing priorities, such as food and shelter needs [12,14,15]. Homeless people are less likely to have a family doctor, compared to the housed population [16]. Their odds of having a family doctor decrease with time passed on the street [14]. A study led in Marseille (France) showed that when homeless people fell ill, the general practitioner is only the fifth solution to turn to (after doing nothing or going to emergency) [17]. Their health care pathways are marked by high rates of acute health care use (emergency department visits), and inpatient admission to hospital [12]. These barriers of access to health care and the inadequate pathways are seen across many countries, with or without universal health care insurance [18,19,20,21]. As a result, homeless people often face acute complications or consult when their health status is already deteriorated. 

According to the World Health Organization, primary health care is “essential care based on practical, scientifically sound and socially acceptable methods and technology made universally accessible to individuals and families in the community (…) It is the first level of contact of individuals, the family, and the community with the national health system” [22]. Several studies showed that the integration of primary care in the management of homeless people improved the diagnosis and the treatment of chronic diseases, improved the care experience for homeless people, and reduced the emergency and inpatient hospital admissions [23,24]. However, it is necessary to deepen the knowledge about programs in primary care which could be useful for homeless people. Some high-quality literature reviews already evaluated interventions to improve health or access to health care for homeless people [12,25,26,27,28,29,30]. The interventions presented in these reviews were various and only a few of them described or evaluated primary care organizations or interventions. The lack of high-quality studies led by programs in primary care for homeless people explained why these systematic reviews, which included only moderate and high-quality articles, contain only a few studies that focus on this topic.

In this review we aimed to describe the main characteristics of primary care programs (organizations or interventions) that take care of homeless people, and to identify which could be most relevant for taking care of the homeless people.

## 2. Materials and Methods

We performed a literature review to identify articles that described and evaluated primary care programs for homeless people.

### 2.1. Data Sources and Research Strategy

We searched into the MEDLINE, PsycINFO, COCHRANE library, and Cairn.info databases primary articles published between 1 January 2012 and 15 December 2016. The research keywords were:

((“homeless”) AND (“primary health care” OR “health services accessibility” OR “general practice” OR “general practitioner” OR “primary care” OR “family physician” OR “family doctor”)) on MEDLINE, PsycINFO, and COCHRANE.

“Homeless AND primary health care”, “homeless AND general practice”, “homeless AND primary care”, “homeless AND family physician”, “homeless AND health services accessibility”, “homeless AND general practitioner”, “homeless AND family doctor” or the same terms in French, on cairn.info.

We also performed a grey literature search, and we added relative articles as we read the references of the selected articles.

### 2.2. Selection Criteria

We selected primary articles which described and evaluated interventions or organizations in primary care, for youth and/or adult homeless people, in English or French. We did not include the review articles, considered as secondary research articles, but we used the references of these reviews to search other pertinent articles. 

### 2.3. Screening

Two reviewers (Jego Maeva and Abcaya Julien) independently screened titles and abstracts, then full texts if the articles were firstly selected. Articles were firstly selected based on title and/or abstract if they were related to homeless people and seemed to describe and evaluate organizations or interventions in primary care for homeless people. A second wave of screening consisted of obtaining full-text articles and reading them from end to end to determine eligibility. Articles were then selected if they provided a sufficient description and evaluated these organizations or interventions. All the disagreements between the 2 reviewers were discussed at each step of the selection. Due to the lack of high-quality articles and comparative methodologies for evaluation, we voluntarily included all articles even if they were not comparative studies or gave few arguments for evaluation (which could be qualitative data, quantitative data, with or without strict comparative schemes).

### 2.4. Level of Evidence

We chose to include all relevant articles, regardless of their quality assessment, in an exploratory approach. The evidence level of each study included in this review was appraised on the U.S. Preventive Services Task Force (USPSTF) 2008. USPSTF has developed a guide to evaluate studies on clinical preventive services and health promotion, and provide recommendations on these themes [31,32]. The USPSTF uses the following hierarchy of research design:

– I (properly powered and conducted randomized clinical trials/well-conducted systematic review/meta-analysis),
– II-1 (well-designed controlled trial without randomization), II-2 (well-designed cohort or case-control analysis study),
– II-3 (multiple time-series, uncontrolled studies),
– III (opinions, based on clinical experience, descriptive studies or case reports, reports of expert committees).

The USPSTF grades the internal validity of randomized clinical trials, cohort studies, and case control studies on 3-point scales (good, fair and poor), which varies between study designs [33].

### 2.5. Data Extraction and Evaluation

We collected data about the general characteristics of studies (year, country where the study was performed, the kind of primary care concerned, and the kind of homelessness concerned), the method of studies, the description of the primary care programs, the outcomes for evaluation, and relative results. We separated the primary care programs between organizations (describing primary care structures, or models, taking care for homeless people) and interventions (specific interventions for homeless people led on primary care teams or with primary care providers). We described the main characteristics of the interventions and organizations presented in the selected articles. Then we classified these characteristics in main categories, as a descriptive thematic analysis. Secondarily, we synthetized the main results about the evaluation of each intervention or organization. We classified these results as follows:

– Positive or negative effects (when comparisons were performed and statistical tests were available),
– Seem to be positive or negative (when studies used a qualitative method, or descriptive data for evaluation, or a comparison without statistical test was available).

## 3. Results

### 3.1. Selection Process and Baseline Characteristics of Eligible Studies

**Figure 1:**
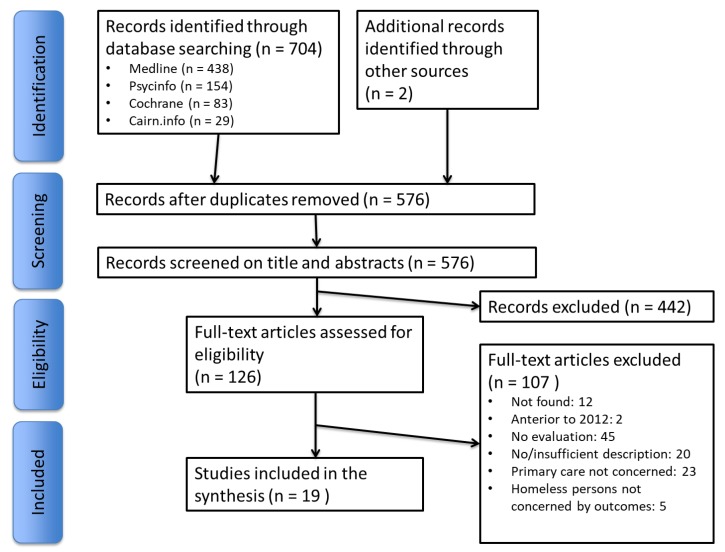
Flow diagram.

The literature search identified 704 articles, and 2 more articles were added with complementary research. After selection process, this literature review included 19 articles [34,35,36,37,38,39,40,41,42,43,44,45,46,47,48,49,50,51,52] (Figure 1).

Most of the selected articles described and evaluated programs led in the USA (12), Canada (3) or the United Kingdom (2). The sample size ranged from 45 to 3543 patients. The level of evidence according to the U.S. Preventive Services Task Force (USPSTF) 2008 classification was various, going from I (higher level, with 2 randomized controlled trials) to III (lower level, concerning 6 studies, with qualitative studies, or low-quality material for evaluation). The internal validity of the studies was mostly fair and poor. Studies included youth, adult and veterans homeless with or without mental illness or substance abuse. Most of the homeless people lived in the street or in emergency or temporary sheltered accommodations. Primary care providers were mostly general practitioners, or nurses. The other primary care providers considered on the studies were non-specified paramedical providers, dentists and medical students. The target areas of the projects raised the following subjects: connected health, access, continuity and quality of care (care pathway), perception of health care by homeless and provider, social issues (with housing status) and training of medical students (Table 1).

### 3.2. Components of Primary Care Programs for Homeless People

Programs (organizations and interventions) in primary care for homeless people often included various components among organizational aspects, models and actions for health care, strategies for enhancing access and/or continuity of care, and non-medical actions. These programs involved mostly team-based, multidisciplinary and/or integrated care. Paramedical primary care providers (nurse managers or public health nurses) were often involved in the multidisciplinary teams. The primary health care models were dominated by patient-centered approach, community health, and care management. The programs often associated case management and patient support, social management, and proposed on-site basic needs. Many programs proposed co-located services between primary health care services, mental health care services, social support, and/or other services, in various combinations. Outreach strategies and low-threshold access were the most used strategies to enhance access to health care. Some programs used an electronic health record to enhance continuity of health care (Table 2).

### 3.3. Effectiveness of Primary Care Organizations for Homeless People

We isolated 13 articles which evaluated primary care organizations for homeless people [34,35,37,38,39,40,41,42,43,44,45,46,47] (Table 3). Most of the articles (9) evaluated tailored organizations for homeless people (pluriprofessional clinics, paramedical clinics and shelter-based clinics). Two studies compared pluridisciplinary clinics with different levels of tailoring (from mainstream to tailored care). Two other studies evaluated non-tailored primary care organizations (integrated primary care of patient-centered medical homes) collaborating with a tailored program for homeless people (housing first program). The organizations had mostly positive outcomes, especially on experience and satisfaction of care by homeless, staff and students, social and housing status, access to and use of health care services. Positive effects on experience and satisfaction of care have been described when: the structures were most tailored [43,44,45], had a friendly atmosphere [34], and offered on-site basic needs [34]. Many studies highlighted favorable outcomes on access to and use of health care [37,38,39,40,41,42], with an increasing of health care visits, and a better use of health care services (mental, primary health care, and others). For example, in a homeless primary-care-based medical home and in patient-aligned care teams, the number of homeless patient visits increased from 20 (before inclusion) to 185 (after inclusion) for the 47 patients included [37]. However, in one study that evaluated a partnership between a non-tailored patient-centered medical home and a housing first program, the on-site primary care physician was the personal physician for only 70 clients among the 183 housed clients and 50% did not have a known primary care provider [47]. In this study, the primary-care provider from a non-tailored medical home performed on-site visits, on-demand from rehoused patients, and homeless people could choose their primary care provider to be another general practitioner. O’Toole et al. analyzed several Homeless “patient-aligned care teams” [35]. These homeless “patient-aligned care teams” consist of a primary care-based, interdisciplinary team model constructed on the principles of patient centeredness, a team-based, whole-person orientation to longitudinal care, and active communication and coordination among providers. The high-performing sites were significantly more likely to track housing status in the clinic notes, to have more than 50% full-time equivalent staffing of a clinic nurse, to have a primary care provider, to offer social services and supports embedded in the clinic, to offer food assistance or clothes on site, and to participate in community events or provide community outreach. 

The impact on care pathways highlighted mostly a reduction of emergency department visits and hospitalizations for some studies [35,36,38], but persistent higher rates of emergency department use for homeless persons compared to persons who have a home were found [38].

The main limits concerned the accessibility of services, a limited capacity to enhance continuity of care, expressed difficulties for the staff to develop multidisciplinary collaboration, and to use electronic health records. 

A detailed description of organizations in primary care and their components is available in Supplementary Material (Table S1).

### 3.4. Effectiveness of Primary Care Interventions for Homeless People

We isolated 6 articles that evaluated primary care interventions for homeless people [36,48,49,50,51,52] (Table 4). The first study (Project Renewal) involved an alcohol treatment intervention based on a chronic care model. The project was based on a clinic whose primary care providers (physician assistant and nurse practitioner) deal with homeless people. This project showed some positive elements (initiation, retention, engagement in treatment and positive experience of participants). However, the results were not statistically significant for mental and physical health status and neither for housing and median number of drinks by month [48]. 

The second study (Pathway project) consisted of a nurse-led and general practice-led in-hospital intervention. This project underscored socio-economic benefits (housing, money, relationship) but had no statistical effect on readmissions, re-attendance at emergency department, duration of hospitalization, drug and alcohol use [49]. 

The third study (StreetMed) was based on a nurse-led primary health care team. The effects on access to health care, registration with general practitioner, continuity of care, housing, or substance abuse reduction seemed to be positive (but no statistical test was available) [50]. 

The fourth study evaluated outreach interventions that included personal health assessments and brief interventions, and a clinic/health system orientation separately and in combination. The authors tested whether these interventions would increase health-seeking behavior and receipt of health care, compared to usual care [36]. On the “personal health assessment and brief intervention” arm, a research nurse interviewed participants and then provided feedback and a brief intervention. On the “Clinic orientation” arm, participants were transported to the clinic by the research assistant where they were introduced to the clinic team. Depending on patient preference and team availability, they were shown where they would need to go to check in, what the process was for being seen, as well as additional resources available at the clinic or where ancillary services were located. On the “usual care” arm, homeless people received a social worker-administered assessment of homeless history and social needs, a description of homeless programs’ services, and verbal and written descriptions of clinical services, with instructions on how they could access care, where to go, and what processes and procedures were involved. Only personal health assessment/brief intervention associating clinic orientation, and clinic orientation alone demonstrated significant improvement in access to primary health care at 1 and 6 months. 

The fifth study (Health shack project) described an electronic health record model developed for homeless youth. This health record was youth-centered, youth-controlled, internet-based, and confidential. The implementation of this record involved a partnership between a drop-in community agency that provided direct services and shelter to homeless youth, the physicians within an academic medical center, and the software developer. This project was acceptable for the youth and seemed to improve the care behavior of the homeless youth. Some limits of the project were highlighted concerning the lack of continuity of care for the homeless youth and limits on reaching the youth [51]. 

The sixth study (Jail Inreach project) consisted of integrated health care with primary care providers who collaborate with mental health providers, social workers and case managers. This project highlighted social benefits (decreased incarceration, social follow-up post release), and medical benefits (continuity and access to health care) [52]. 

More detailed descriptions of interventions are available in supplementary material (Table S1).

## 4. Discussion

### 4.1. Main Results

Primary care programs for homeless people involved mostly team-based, multidisciplinary and/or integrated care. Many programs proposed co-located services between primary health care services, mental health care services, social support, or other services, in various combinations. Most of the programs had strategies to enhance access to health care, dominated by outreach strategies and low threshold access. The primary health care models were dominated by patient-centered approach, community health, comprehensive approach, and care management. The programs often associated case management with patient support and social management, and proposed on-site basic needs. The positive outcomes of these programs concerned mostly the satisfaction obtained by the homeless people through the health care system and the experience that the staff and the students gained. Other frequent positive outcomes were: the change of the social status, the housing status and the access and use of the health care services. The accessibility of services, a limited capacity to enhance continuity of care, the expressed difficulties for the staff to develop multidisciplinary collaboration, and the use of electronic health records were the main limits identified on the programs. The high-performing primary care programs were significantly more likely to track housing status, to include a clinic nurse in staff, to offer on-site social services and basic needs, and to be engaged in the community. Tailored primary care organizations for homeless people led to significant better experience of care for homeless people with or without mental illness, compared to mainstream clinics. Clinic orientation (associated or not with personal health assessment and brief intervention) significantly improved access to health care for homeless people. 

### 4.2. Integrating Care and Non-Medical Services to Face the Complex Needs of Homeless People

In this literature review, each primary health care program combined medical, paramedical and non-medical components in various combinations. The medical components were dominated by multidisciplinary approaches, including team-based approaches, integration of health care, coordination, and active collaboration among providers. In the 2000s, coordinated treatment programs for the management of mental illness and substance abuse disorder showed better health outcomes than usual care alone [25]. Fazel et al. recommended that health services focus on the identification and management of somatic diseases (especially infectious diseases and diseases of old age, considering the ageing of homeless people in the USA), and mental illnesses. They also recommended integration “across medical specialties, particularly with treatment providers for addictions, and also to address unmet social and housing needs” [1]. In a qualitative study led in Marseille regarding general practitioners, maintaining a stable follow-up was a major condition for GPs to contribute effectively to the care of homeless people This study identified some key answers to improve the continuity of the health care for homeless people: active outreach, individual support, multidisciplinary and team-based approach, and enhanced social management involving closer relations between medical actors and social workers [53]. These elements lead us to conclude that primary health care programs which develop multidisciplinary care as teams, integration of health care between somatic and mental health, and integration between health and social services are more suitable to address homeless patients’ complex needs. 

### 4.3. Resolving the Debate between Tailored and Non-Tailored Programs to Take Care of Homeless People?

Wright et al. described three main frameworks that provide primary health care to homeless people [54]: the first is mainstream general practice (most of the time with a “special interest” on homeless people), the second is a “specialized” general practice that registers only homeless people (tailored programs for homeless people), and the third is primary health care provided for homeless people in hospital secondary care.

As found in this study, the recent literature has shown that tailored programs were more effective, provided more appropriate care and a better experience of care than the standard primary health care [24,44,55], yet many limitations of tailored programs were described in the literature. Firstly, these programs sometimes have insufficient resources to meet such high-level care requirements [56]: this limit concerns mostly non-governmental associations involving volunteer workers. Secondly, for some authors, it can cause a feeling of humiliation, and engender a decreased seeking of care by their patients [57]. Lastly, Lester et al. explained that parallel pathways could reinforce the feeling of exclusion of the homeless, and enhance the ghettoization of their care [58]. Those authors proposed a better model that might combine specialized and mainstream primary care services. In such a model, they recommended that homeless people could register with a specialized homeless practice when they are in crisis. Once their urgent needs have been met, homeless people could then be helped to permanently register within mainstream general practice. This model creates a bridge between separation and integration of tailored and non-tailored services for the homeless. Only 2 programs in this study involved non-tailored primary care collaborations with tailored structures. Even if the results seemed positive, in one study the lack of a primary care registration was highlighted as a limit. The lack of information about non-tailored structures collaborating with tailored structures makes us unable to say for sure that a model with a close collaboration between non-tailored primary care centres and the tailored structures can be a fair transitional solution.

### 4.4. Other Efficient Interventions Should Be Associated to Primary Care Programs for Homeless People

The literature reviews offered some evidence suggesting that the orientation to clinic services available either alone or combined with the outreach improves the access to primary care providers among adults who are homeless, without serious mental illness, and living in urban centres [12]. Other interventions which do not concern primary care seem to improve access to health care, health status, or housing status for homeless people. An international program that has the main activity of housing the homeless people and associate case management and social support (“Housing first”) improved outcomes for homeless people with serious mental illness [24]. Case management and assertive case management were also identified as being effective in improving psychiatric symptoms, and decreasing substance use for homeless people with substance abuse issues [25]. Improving the global health of homeless people cannot depend only on primary care programs. It should associate other interventions, acting on housing, social support or specific health needs (mental health, substance abuse, for example). 

### 4.5. Strenghts and Limitations of This Study

This literature review gives a comprehensive and a deep understanding of the primary care programs developed for homeless people. The other recent literature reviews targeted only the programs evaluated in high-quality quantitative and comparative studies. In these reviews, if the included articles and the review’s method were of high quality, the main and only setback was the low number of articles that could be included [12,24,25,26]. We then decided to include high- to low-quality studies, and all the types of study (from clinical trial to qualitative studies). As a result of this choice, the internal validity of our results was limited. Indeed, only 3 studies (1 comparative survey and 2 randomized trials) were classified with good internal validity. That is why this literature review gives little evidence over the efficiency of the primary care programs that were designed for homeless people.

We detailed in this review the main components of the primary health care’s interventions and organizations. Nevertheless, the efficiency of the primary care programs for homeless people may also depend on the different systems of health care [59]. It would be interesting to explore how these programs work and what is their impact considering the various global health care systems of each country. 

The programs described in this review often involved multidisciplinary partnerships or participative approaches to community care. The studies included also described participatory research actions, which is the co-construction of research through partnerships between researchers and people affected by and/or responsible for action on the issues under study [60,61]. We did not use terms relative to community-based and/or participative approaches in this review. It would be interesting to explore the field of research for homeless people.

## 5. Conclusions

The primary care programs included in this literature review used mostly team-based approaches, multidisciplinary and/or integrated care. They often proposed co-located services between somatic, mental health and social support, and they tried to answer the specific needs of homeless people. This review isolated some characteristics that have proven effective: tailored primary care organizations for homeless people, clinic orientation (that improved the access to the health care system for homeless people), multidisciplinary team-based models, including primary care physicians and clinic nurses, integration of social support (housing support, co-located social services, and on-site basic needs availability), and engagement in community health. The majority of the studies described tailored primary care programs; therefore, it is necessary to evaluate more non-tailored primary care programs that collaborate with tailored structures in order to be able to take into discussion the efficiency of such programs.

## Figures and Tables

**Table 1:** General characteristics of included studies.

Reference	Country	Study Type	Effectives and Population (ETHOS ^1^ Categories of Homelessness)	Target Areas	Level of Evidence/Validity ^2^
Dang et al., 2012 [51]	USA	Qualitative study	Effectives: 149 Homeless youth (ETHOS non-specified)	Connected health	III (NC ³)
Uddin et al., 2012 [40]	Bangladesh	Comparative survey (before/after)	Effective: 804 Homeless people (living rough)	Access to health care/health status/experience of care	II-3 (fair)
Simons et al., 2012 [39]	UK	Comparative survey (Here and elsewhere)	Effectives: 350 (150/250 for 2 clinics) Homeless people (living rough, in accommodations for the homeless, temporary or non-conventional structures) + rehoused homeless people	Access to health care/Continuity of care/Health needs meet	II-3 (poor)
Held et al., 2012 [52]	USA	Comparative survey (before/after)	Effectives: 150 People due to be released from institutions (jail)	Social outcomes/access to health care/Community integration	II-2 (fair)
Omori et al., 2012 [43]	USA	Qualitative study	Effectives: unknown Homeless youth/families/adult (living in accommodation for the homeless)	Experience of care/medical training	III (NC)
Weinstein et al., 2013 [47]	USA	Mixed study	Effectives: 183 (quantitative)/11 (qualitative) Homeless people with mental illness (living rough, in a night shelter)	Quality of care/Public health missions accomplishment	III (NC)
Rowan et al., 2013 [34]	Canada	Mixed study	Effectives: 72 (quantitative)/13 (qualitative) Homeless youth (living rough, in a night shelter, in insecure accommodation, in temporary or non-standard structures, unfit housing)	Experience of care/Continuity of care Program assessment	III (NC)
Kertesz et al., 2013 [44]	USA	Comparative survey (5 centres)	Effectives: 601 Homeless people, veterans and not veterans (living rough, in a night shelter, in temporary or non-standard structures)	Experience of care	II-2 (good)
Patel et al., 2013 [37]	USA	Comparative survey (before/after)	Effectives: 47 Homeless veterans (ETHOS non-specified)	Access and continuity of care/care pathway/Housing	II-3 (poor)
O’Toole et al., 2013 [38]	USA	Case—control study + cohort analysis	Effectives: 233 (127 cases/106 controls) Homeless veterans (living rough, in emergency accommodations, in accommodations for the homeless, temporally with families or friends)	Access to health care/screening	II-2 (fair)
Campbell et al., 2013 [42]	Canada	Qualitative study	Effectives: 45 Homeless people (in accommodation for the homeless)	Experience of care/Access to health care	III (NC)
Carson et al., 2013 [46]	USA	Retrospective study	Effectives: 123 Chronic homelessness and serious mental illness (ETHOS non-specified)	Quality of care	III (poor)
Lamb et al., 2014 [50]	UK	Case study/literature synthesis	Effectives: 86 Homeless people (living rough, in night shelter, in temporary or non-standard structures)	Access to health care/Housing	III (NC)
Chrystal et al., 2015 [45]	USA	Comparative survey (5 centers)	Effectives: 366 Homeless people with mental illness, veterans and non-veterans (ETHOS non-specified)	Experience of care	II-2 (fair)
O’Toole et al., 2015 [35]	USA	Randomized controlled trial	Effectives: 185 (39, 44, 62, 40 for 4 arms) Homeless veterans (living rough, night shelter, in accommodation for the homeless, or transitional housing)	Access to health care	I (good)
Stergiopoulos et al., 2015 [41]	Canada	Quasi-experimental study	Effectives: 140 (70 in both arms) Homeless with mental illness (living in shelter)	Housing/Social outcomes/Access to health care/quality of care	II-2 (fair)
Upshur et al., 2015 [48]	USA	Randomized controlled trial	Effectives: 82 Homeless women with drinking problems (ETHOS non-specified)	Alcohol abuse/Continuity of care/health status/housing status	I (poor)
Hewett et al., 2016 [49]	UK	Randomized controlled trial	Effectives: 410 (206/204 for 2 arms) Homeless people (living rough, in emergency accommodation, due to be released from institutions/living in temporary or non-conventional structures)	Care pathway/accommodations at discharge/quality of life	I (good)
O’Toole et al., 2016 [36]	USA	Comparative survey (before/after)	Effectives: 3543 Homeless veterans (ETHOS non-specified)	Access to Health care/care pathway	II-2 (fair)

^1^ European Typology on Homelessness and Housing Exclusion [2]; ^2^ According to the U.S. Preventive Services Task Force USPSTF 2008 classification (internal validity criteria are available for Randomized clinical trials, cohort studies and case-control studies); ³ NC: not concerned.

**Table 2:** Main components of primary care programs (organizations or interventions) for homeless people.

Components of Programs (Refs.)	Nb (%)	Details
**Organizational themes**		
Multidisciplinary care [34,35,36,37,38,39,41,46,47,51,52]	11 (56%)	
Team-based approach [35,37,38,39,41,44,45,46,47]	9 (47%)	
Active collaboration among providers [35,37,48]	3 (16%)	Coordination, communication between providers, collaborative care
Integrated care/services [34,36,39,41,43,45,46,47,50,52]	10 (53%)	Primary care and mental health care; Health care and social care ± housing support; whole integration (primary care, mental health care, social and housing support)
Paramedical primary care [36,37,38,40,46,49,50,51]	8 (42%)	Nurses: Case manager, research nurses, public health nurses/others (dental, other paramedics non-specified)
Staff training [35,44,45,48]	4 (21%)	Homeless-focused training, primary care providers training for interventions
Co-located services [34,35,38,39,44,46,52]	7 (37%)	Various combinations with Primary-care; Mental health; Drop-in center; Health care and social ± housing; other: paramedical, family planning, dental…
Multi agencies or interprofessional partnerships [34,44,47,49,51]	5 (26%)	Shelters, homeless organizations, medical center, housing first program, public health center, academic center, multi-agencies care plans
Primary care centres linkage [41]	1 (5%)	
Training missions [34,41,42,43]	4 (21%)	Teaching clinic, linkage with teaching hospital, medical students, dental students, academic linkage
Public health concerns [47,51]	2 (11%)	
Shelter-based care [41,43,46]	3 (16%)	
Hospital in-reach team [49]	1 (5%)	
**Models and actions for health care**		
Community health [35,39,41,49,52]	5 (26%)	Collaboration with community agencies, integration with community services, linkage
Preventive care and screening [40,43]	2 (11%)	
Care management [35,36,38,40,43,46,48]	7 (37%)	Coordination of care, addressing, intensive care management, linkage to primary or specialized care
Coordinated care [37,38,39,40,41,42,43,46,48]	9 (47%)	
Comprehensive care [37,52]	2 (10%)	
Patient-centered approach [35,36,39,47,48,50]	6 (32%)	Patient-centered care, Health assessment, goal setting, whole patient orientation, holistic care, case-based approach, self-management support
Health education for users [36,39,40,43,47]	5 (26%)	
Brief intervention [36,48]	2 (11%)	
**Tools for enhanced continuity of care**		
Personal card (paper) [40]	1 (5%)	
Electronic health record [38,41,48,51,52]	5 (26%)	Shared or not
Monitoring systems [46]	1 (5%)	
**Other non-medical actions**		
Case management/patient support [36,37,41,49,50,52]	6 (32%)	including accompaniment
On-site basic needs availability [34,35,36,38,45]	5 (26%)	
Social management [35,36,38,42,43,46,50]	7 (37%)	Social support with social workers/social management by physician
Well-being actions [43,48]	2 (10%)	Fun events, gifts, wellness counseling
Peer-workers [44,45]	2 (10%)	
**Access to care awareness**		
Information of users [36,39,40]	3 (16%)	
Low threshold access [35,38,39,42,43,45,52]	7 (37%)	Free care, walk-in, with or without appointments, emergency appointments, open access, on-demand
Outreach [35,39,40,43,44,45,47,50]	8 (42%)	Active outreach, mobility, outreach within community, street or shelters outreach
Friendly atmosphere [34,40]	2 (11%)	Decorations for local of vans

**Table 3:** Effectiveness of primary care organizations for homeless people.

Primary Care Organizations	Ref.	Method for Evaluation and main Results
**1/Tailored organizations**		
Medical and dental primary care (pluriprofessional)	Rowan et al. [34]	**Method:** qualitative (interviews with providers, focus groups with youth) **Seem to be positive (qualitative data):** – satisfaction by youth (atmosphere, location, free things provided, free care) and staff (perceived role, relation with youth) **Seem to be negative (qualitative data):** – Staff (difficulties for interdisciplinary collaboration and electronic medical record use) – Youth (limited accessibility of services)
Homeless primary-care-based medical homes and patient-aligned care teams (PCMH-PACT)	O’Toole et al. [35]	**Method:** compared pre- and post-enrollment (at 6 months) use of health services/stratified site (high/moderate/low performing) then compared their parameters. **Seem to be positive (no statistical test):** – care pathway: at 6 months, ↓ Emergency Department visits from 19%/Hospitalizations from 34.7% **Characteristic of high-performing sites:** more likely to: track housing status *, have >50% full-time equivalent staffing of a clinic nurse *, have primary care provider *, have social services and supports embedded in the clinic *, offer food assistance on site *, have a clothes pantry *, participate in community events * or community outreach *
	Patel et al. [37]	**Method:** pre-post evaluation with univariate analyses **Positive:** – social management: ↑ number of appointments with social workers (149 for 27 patients before versus 371 for 36 patients after *) – access to health care: on 47 patients, 185 visits for H PACT after versus 20 before *
	O’Toole et al. [38]	**Method:** comparison between homeless and non-homeless patients (at baseline and at 6 months)/comparison for homeless people subgroup with pre–post evaluation. **Positive:** – Use of health care (during first 6 months): mental health services (88% for H PACT versus 43.4% for non-homeless *), substance abuse treatment services (37.8% for H PACT versus 7.5% for non-homeless *) – emergency department use (before/after): ↓ for homeless veterans who accessed primary care at higher rates * or who used specialty and primary care *. **Negative:** emergency department use higher during first 6 months for H PACT *
Dental clinic	Simons et al. [39]	**Method:** comparison between Mobile dental service (MDS) and dedicated dental service (DDS) (no test)/bivariate analyses to assess the relationship between service use and the overall outcomes of care **Seem to be positive:** – Access: better for mobile clinic: more rough sleepers accessing the MDS mobile dental service (10%) compared to the DDS (dedicated dental service) (1%). – Efficiency improvement: lost clinical time ↓ between 2009 and 2011 **Seem to be negative:** – Continuity of care: 36.7% patients lost after the first appointment (more from the MDS than the DDS). Only 27.8% of patients completed a course of treatment.
Paramedics-led clinic	Uddin et al. [40]	**Method:** mixed-method approach combining both quantitative and qualitative techniques **Positive:** – Morbidity: ↓ in both models for women and men street-dwellers * – Use of health care services: ↑ in both models for women and men street-dwellers * – Health behaviors/prevention: ↑ family-planning method use in both models * **Seem to be positive:** perception of street-dwellers and service providers
Shelter-based clinics (collaborative care models)	Stergiopoulos et al. [41]	**Method:** Multivariate regression models to compare study arms **Positive:** – Community functioning: scores at 6 months and 12 months higher than at baseline * for shifted outpatient collaborative care model (SOCC) and integrated multidisciplinary collaborative care (IMCC). – Health services utilization: improved, with more effect for SOCC [↓ emergency department visits at 6 and 12 months (Odd Ratio (OR) = 0.51 IC 95% (0.30–0.87) and OR = 0.48 IC 95% (0.26–0.90) * respectively/↓ overnight hospital visits at 6 and 12 months (OR = 0.45 IC 95% (0.26–0.79) and OR = 0.33 IC 95% (0.17–0.63) *, respectively)/↑ Community physician visit in the past 30 days at 12 months (OR = 2.07 IC 95% (1.14–3.74) *]
Shelter-based clinics (Student-run clinic)	Campbell et al. [42]	**Method:** qualitative (individual and group semi-structured interviews with stakeholders) **Seem to be positive:** Education purpose/Better relationships perceived/Feeling that students were in a good position to help make referrals to specialists’ care (non-client participants)/Increased capacity to access (two more than the physician would see when working without students)/co-location in shelter well perceived **Seem to be negative:** Access (only for a subset of the homeless population/Limit for providing continuity of care)/Student knowledge and experience as a limit
	Omori et al. [43]	**Method:** descriptive and qualitative data (non-specified) **Seem to be positive:** – Patients’ benefits: high satisfaction ratings. – Students’ benefits: improved clinical skills, improved attitudes towards caring for the homeless, promotion of future volunteerism, increased patient advocacy skills, improved knowledge of systems-based practice principles, resource allocation, cost containment, increased interaction amongst the different levels of medical students, continuity of care with patients as being extremely helpful and rewarding. – Physicians’ benefits: positive feeling by working with underserved patients + teaching medical students
**2/Tailored compared to mainstream organizations**		
Pluriprofessional primary care clinics	Kertez et al. [44]	**Method:** survey-based comparison **Positive for tailored sites:** more positive experiences at the tailored non-Veterans Affairs (VA) site than at the 3 mainstream VA sites. Adjusting for patient characteristics, differences remained significant for the relationship and cooperation */Unfavorable experience 1.5 to 2 times more common at the mainstream VA sites compared with the tailored non-VA site *
	Chrystal et al. [45]	**Method:** survey-based comparison **Positive for tailored sites:** site of care with tailored sites and Mainstream A (the one with some tailoring) obtained more favorable scores for experience of care (mean = 3.14 in tailored non-VA/3.05 in tailored VA/3.06 in mainstream VA A/2.96 in mainstream VA B/2.93 in mainstream VA C *
**3/Non-tailored organizations collaborating with tailored structures**		
Integrated health care: Housing first with integrated primary health care	Weinstein et al. [46]	**Method:** retrospective data, comparison between housing first and integrated care subgroup (no test) **Seem to be positive:** – Quality of care/screening: Receipt of Recommended Quality Assurance Measures (higher in integrated care subgroup compared to Housing first participants, for every categories)
University patient-centered medical home	Weinstein et al. [47]	**Method:** auto-rating on Likert values **Seem to be positive (no comparison):** partnership functioning as an integrated person-centered health home. Addressed the public health objectives according to “10 Essential Public Health Services” **Seem to be negative (no comparison):** The on-site primary care physician was the personal physician for 70 clients on the 183 housed clients; 50% did not have a known PCP

* *p* < 0.05.

**Table 4:** Effectiveness of interventions in primary care for homeless people.

Interventions	Ref.	Brief Description of Interventions	Method for Evaluation and Main Results
**Interventions involving tailored primary care**			
Project renewal: alcohol treatment intervention based on chronic care model	Upshur et al. [48]	Homeless clinic with primary care providers (Doctors of Medicine, physician assistants and nurse practitioners)	**Method:** randomized controlled trial, comparison between average number of drinks with linear regression **Positive:** initiation, engagement and retention in treatment: ¼ of intervention women versus >50% women who did not use this service at 3 months *; 76% group intervention versus 44% in usual care had three or more contacts with any substance use treatment *; 75% versus 47% at 6 months *. Mean of 12.1 total visits (with case manager) versus 6.2 for usual care *. **Seem to be positive:** experience of participants: Most were positive about the intervention experience (qualitative data).
Pathway project: nurse-led and General Practitioner-led in-hospital intervention	Hewett et al. [49]	Intervention from primary care providers (nurse and general practitioners in hospital	**Method:** randomized parallel-arm trial, adjusted regression model for comparison **Positive:** – Housing: 14.6% of patients in the control arm were street homeless at discharge compared with 3.8% of patients in the intervention arm (odds ratio = 0.14) * – Money: Intervention ↑ score for money (mean 3.85 at baseline versus 5.21 at follow-up) * – Relationship: intervention ↑ score for relationships (mean 4.79 at baseline versus 5.68 at follow-up) *
Streamed: nurse-led team project	Lamb et al. [50]	Nurse-led team primary care project	**Method:** descriptive data about effectiveness outcomes + case study **Seem to be positive (no statistical test/case study):** – Access: The number of patients registered with a general practitioner increased from 17 to 48 (on 86 patients), and the percentage of rough sleepers or those without secure accommodation reduced from 68 to 37% (p unknown) – Multiple positive consequences described but on a case study treating about 1 case (joe, 54 years old) (access, continuity, substance abuse reduction, housing project . . .)
Outreach interventions	O’Toole et al. [36]	Outreach interventions	**Method:** randomized controlled trial, uni- and bivariate analyses for comparison (ANOVA, Cox proportional-hazards regression survival analysis) **Positive:** access to primary care at 6 months improved **only for CO (clinic orientation) and PHA/BI (Personal Health assessment/Brief intervention) +CO** (cox regression using usual care as reference): – PHA/BI + CO → At 1 month, 77.3% accessed primary care versus30.6% in usual care arm/At 6 months, 88.7% accessed primary care versus 37.1% for Usual care, hazard ratio = 3.41 * – CO-only: At 1 month, 50% versus 30.6% in usual care arm/At 6 months, 80% versus 37.1% in usual care, hazard ratio = 2.64 *
**Interventions involving non-tailored primary care but collaborating with tailored structure**			
Health shack: web-based personal health information system	Dang et al. [51]	Partnership with a tailored structure drop-in community agency for homeless, the software developer, and physicians within an academic medical center, and the software developer.	**Method:** descriptive data and mostly qualitative evaluation **Seem to be positive (qualitative data):** – Acceptability for youth: youth felt positive about enrolling in Health shack and were comfortable using this technology. They denied concerns about confidentiality after meeting with the public health nurses and being informed about confidentiality laws. – Care behavior: many Health shack participants voluntarily returned to see the public health nurses to discuss confidential health issues. **Seem to be negative (qualitative data):** – Follow-up: loss of contact with some potential enrollees. – Reach: youth referred by Health Ambassadors sometimes did not show up as planned, and attempts to reach youth were unsuccessful
Jail Inreach Project	Held et al. [52]	Integrated health care: primary care providers collaborating with mental health providers and social workers/case managers	**Method:** comparison with analyses of variance test (univariate) **Positive:** Outcomes relative with incarceration (county Jail ↓/number of charges per person ↓/total number of average felonies per year ↓/total average misdemeanors ↓) * Seem to be positive (no test): Continuity of care/access: >50% of jail releases referred to the Jail Inreach Project continued with services after returning to the community, VS <33% of releases not participating in the program. 65.7% of the study sample who were approached by a case manager while incarcerated and agreed to participate were linked to community health and social services post release via the Jail Inreach Project.

* *p* < 0.05.

## Supplementary Materials

The following are available online at http://www.mdpi.com/1660-4601/15/2/309/s1, Table S1. Description of programs in primary care caring for homeless people.
