# Supplementary material for: Improving Health Care Management in Primary Care for Homeless People: A Literature Review
Source: Int J Environ Res Public Health. 2018 Feb 10;15(2):309. doi: 10.3390/ijerph15020309 (PMC5858378; doi:10.3390/ijerph15020309)
Supplement: Supplementary file 1 [file ijerph-15-00309-s001.pdf]

International Journal of Environmental Research and Public Health

MDPI

Supplementary Materials: Improving Health Care Management in Primary Care for Homeless People: A Literature Review

Maeva Jego [1,2,*], Julien Abcaya [2], Diana-Elena Ștefan [3], Céline Calvet-Montredon [2] and Stéphanie Gentile [1]

Table S1. Description of programs in primary care caring for homeless people

Study	Kind and description of programs	Main characteristics of programs
	Medical and dental primary care	
Rowan et al. An innovative medical and dental hygiene clinic for street youth: results of a process evaluation. 2013. [34]	Clinic housed at a drop-in center. Open 4 afternoons per week, with different providers each having a designated day for service provision. Other project partners : Public Health center, Community Health Centre, family health network that provided physician and resident services and College Dental Hygiene Program.<br>Basement location: two examining rooms, waiting room, washroom and small storage area. Decorated in a youth friendly atmosphere with bright paint, health education posters, baskets of free condoms and sunscreen.	Co-located services (drop-in centre) / multi agency partnerships / multidisciplinary care / training mission / on-site basic needs availability / friendly atmosphere
O'Toole et al. Tailoring Care to Vulnerable Populations by Incorporating Social Determinants of Health: the Veterans Health Administration's "Homeless Patient Aligned Care Team" Program. 2016. [35]	Homeless Patients Aligned Care Team (H-PACT) and homeless medical home model<br><br>Enhanced low-threshold access to care with open-access, walk-in capacity, flexible scheduling, and clinical outreach to homeless people on streets, in shelters, and in community locations; Integrated services (mental health services and primary care services are located close to each other, and providers from both services are involved in patients' health care plan) + sustenance needs available at the same location; Intensive health care management integrated with community agencies, Ongoing staff training and development of homeless care skills, Data-driven, accountable care processes.	Integrated care (primary care/mental health care/social care) / team based approach / multidisciplinary care / patient-centered approach / whole-person orientation / care management / coordination of care / / community health / co-located services (primary care/mental health) / on-site basic needs availability / active collaboration among providers / low-threshold access (open access, walk-in, flexible) / outreach / homeless-focused staff training
Patel et al. Complex Care Management to Decrease Emergency Department Utilization: A Case Study of the Homeless Patient Aligned Care Team Demonstration	Homeless Patient Aligned Care Team (H-PACT) on Patient Centered Medical Home (PCMH)<br><br>The PCMH is a patient-driven, team-based approach that delivers efficient, comprehensive and continuous care through active communication and coordination of healthcare services. PACT in PCMH implemented at veteran homeless centre. PACT provides accessible, coordinated, comprehensive, patient-centered care, and is managed by	Coordinated care / multidisciplinary care/ team based approach / patient-centered approach / case management / comprehensive primary care / active collaboration among providers

Project at VA Greater Los Angeles Healthcare System. 2013. [37]	primary care providers with the active involvement of other clinical and nonclinical staff (nurses as cases manager/ licensed vocational nurse).	
	**Homeless Patients Aligned Care Team (HPAC-T) and medical home model**	
O'Toole TP et al. New to care: demands on a health system when homeless veterans are enrolled in a medical home model. 2013. [38]	**P**rovidence Veterans Affairs Medical Centre: primary care providers assigned to 8 general medicine teams (PACTs) or 4 special population PACTs (geriatrics, women's heath, serious mental illness and homeless) / Nurse case manager / Specialty care (cardiology, endocrinology, pulmonary, podiatry, optometry, general surgery and surgery subspecialties) / Mental health /Addictions services.<br>Homeless PACT: provide on-demand or walk-in care with no appointments needed on designated clinic days. Homeless services (housing assistance, vocational services, benefits, social work) are collocated in the homeless PACT, as well as other support services (clothes pantry, meals). Patients are redirected to another team or available provider if necessary. Both the general medicine and homeless teams had assigned nurse case managers, ancillary team support, and used electronic medical record. They also received monthly clinical performance reports and participated in departmental quality improvement initiatives.	Multidisciplinary care / team based approach / co-located services (housing/social/primary care and specialty care/mental health/addiction)/ on-site basic needs availability / paramedical primary care / coordinated care / electronic medical record / case management (addressing) / low-threshold access (walk-in, on demand)
Simons D. et al. Developing dental services for homeless people in East London. 2012 [39]	Dedicated dental primary care clinics<br><br>- Dedicated homeless MDS (mobile dental service): locations chose with local stakeholders, continually reviewed for client uptake. Combination of emergency appointments, drop-ins, check-up slots and treatment appointments. Information provided.<br><br>- Dedicated homeless fixed site health center with a DDS (dedicated dental service) : multidisciplinary 'one-stop shop' – with general medical services, podiatry, dentistry, psychologist, health visitors, family planning and blood clinics, a Blood Born Viruses team, substance misuse clinics, mental health clinics and alcohol services etc., all delivered together at a single physical location. Case based approach, with the physical health, mental health and substance misuse problems of each individual patient/client being dealt with in a coordinated way. By appointment only in theory, but in practice also operates on a « walk-in » basis. Clients attracted by means of street outreach / visits to hostels/day centres.	Outreach (active outreach) / community health (collaboration with community agencies) / outreach (mobility) / low-threshold access (with or without appointments) / information/education of users / outreach (street/active outreach) / co-located services (primary care, substance use, mental health, dental paramedical, family planning services) / multidisciplinary care / team based approach / integrated care (physical/mental/substance misuse) / case based approach / coordinated care

	Paramedics-led clinics (static and mobile satellite clinics)_	
Uddin J et al. Strategies for providing healthcare services to street-dwellers in Dhaka city: Evidence from an operations research. 2012. [40]	Two paramedics engaged in providing primary health care services, who were trained by the experts from a primary healthcare program. The primary healthcare services were provided from the clinic in the evening twice a week (static clinic) and 2 times per week (satellite clinic). Area of actions: General health / Reproductive and maternal health / Family-planning methods /Child health / Expanded Program on Immunization. Referral linkage were made to address nearer health facilities, in government and non-governmental facilities, to refer patients from both static and satellite clinics. A patient card were provided to the clients to keep track in subsequent visits . Decorated van were used for carrying the clinic staff and logistics, for publicity, and for carrying patients from the static clinic to the referral points. Information of street-dwellers was provided about the availability of services, and names and addresses of the clinics and types of services available.	Outreach / preventive care / paramedical primary care / care management (addressing) / continuity of care (card) / information and education of users / friendly atmosphere / coordinated care
	Collaborative care models	
Stergiopoulos V et al. The effectiveness of an integrated collaborative care model vs. a shifted outpatient collaborative care model on community functioning, residential stability, and health service use among homeless adults with mental illness: a quasi-experimental study.2015. [41]	IMCC (Integrated multidisciplinary collaborative care): shelter- based multidisciplinary health team, which adopted an IMCC model of service delivery in partnership with a local teaching hospital. Integrated care: on-site psychiatrist 4,5 days/week, as an integral part of a primary care team : direct patient care + indirect patient discussion and educational support to team members. Shelter staff and health care providers work as a single team and share a common electronic medical record. SOCC (Shifted outpatient collaborative care model): a psychiatric consultant, not linked administratively to the shelter, provides outpatient care in the shelter setting (1/2 day/week: direct patient care and indirect patient discussion and educational support). The consultant psychiatrists share an electronic medical record with select shelter staff. Primary and mental health care are not integrated, and referrals to mental health are initiated by shelter staff. No nursing services on site: such services are accessed through neighboring primary care centers. Other health provider support is obtained, as needed, through referral to other community agencies.	Multidisciplinary care / coordinated care / training mission / shelter-based care / electronic medical record (shared) / case management / primary care centers linkage / integrated care (primary health/mental health/housing) / community health (linkage) / team based approach
	Shelter-based student-run clinic in general practice	
Campbell DJ et al. The role of a student-run clinic in providing primary care for Calgary's homeless populations: a qualitative study. 2013. [42]	Based at a Drop-in and Rehabilitation Centre (homeless shelter). Weekly, during evening clinical hours, a team of 2 students see the patients. They take the history and perform an initial physical examination that is reviewed with the attending physicians. They develop a plan for the patient's care. General primary care is provided to whomever requests clinic services. There is no discrimination on the basis of payment. Patients are seen in the Calgary SRC free of charge, even if they do not have proof of government health insurance. Students also assist patients with logistical arrangements for specialists or supportive care, and provide assistance obtaining relevant insurances or emergency medication payment if required. The clinic schedule is arranged so that there is always one person present in the clinic on any given week who was also present the prior week.	Low-threshold access (free care) / training mission / social management (by physicians) / coordinated care

	Shelter-based student-run clinic in general practice	
Omori et al. The Hawai'i Homeless Outreach and Medical Education Project: servicing the community and our medical students. 2012. [43]	"H.O.M.E. Project": student-run, free clinics at homeless shelters + outreach services with a mobile health van. Education: First year students that participate with H.O.M.E. Project Clinics : they acquire experience (performing intake histories, vital signs), and help in the overall functioning of the clinics + coordinate care for patients. Second year students can become managers : they help to supervise the first year community health students, provide orientations for the third year students and are responsible for keeping track of equipment, supplies, and medications. Third-year -> clerkship experience, Fourth-year --> longitudinal elective as providers. Actions: care for acute and chronic medical problems, health maintenance and prevention, other actions (vaccinations, diagnosis with laboratory (freely for the uninsured), tuberculosis testing or imaging, health education, dental assessments, and free medications (for the uninsured). They arrange patient appointments with specialists, coordinate laboratory and imaging services, collaborate with the shelter social workers. Annual special events for the shelters (Halloween, Christmas, ...), gifts for the children, and , a family fun day that promotes healthy living and family togetherness + monthly excursions with students and teens at the shelters + monthly interactive sessions with the teens on various health and social topics.	Training mission / low-threshold access (free care) / outreach / care management (addressing of specialty care) / integrated care (health/social) / coordinated care / education of users / well-being actions / preventive care / screening
	Pluriprofessional primary care clinics (tailored versus non tailored)	
Kertesz et al. Comparing homeless persons' care experiences in tailored versus non tailored primary care programs. 2013. [44]	1/ Mainstream veterans affair (VA) clinics A and C: Primary Care and mental health in same clinical space<br>2/ Mainstream VA clinic A : primary care in shelters and streets + formal relationships to community shelters<br>3/ Tailored VA : explicit homeless mission / homeless-focused staff training/ Primary Care and mental health in same clinical space<br>4/ Tailored non VA : explicit homeless mission/primary care in shelters and streets / Team design assures continuity from streets/shelter to clinic / formal relationships to community shelters / Homeless-focused staff training / Primary Care and mental health in same clinical space / Linkage to national homeless organizations / Formerly homeless persons in organizational governance / > 10 years explicit homeless mission focus	Co-located services (mental/primary care) / outreach/ homeless-focused staff training / team based approach / peer-workers / multi agency partnerships (community shelters/homeless organizations)
	Pluriprofessional primary care clinics (tailored versus non tailored)	
Chrystal et al. Experience of primary care among homeless individuals with mental health conditions. 2015. [45]	Tailored clinics (VA and non-VA) : dedicated program staff, specialized training of staff, heavy emphasis on walk-in availability, capacity to respond to tangible or competing needs (such as food, washing or clothing), integrated mental health care, and inclusion of homeless individuals in organizational governance. Tailored VA site included co-located mental health and primary care with an emphasis on access and same-day services. Tailored Non-VA site had the most homeless-centric service characteristics, providing outreach care within the community, homeless focused medical and nursing staff, as well as representation of homeless and formerly homeless persons in organizational governance<br>Mainstream sites (VA-A, VA-B, VA-C) : mainstream primary care operations within standard VA clinic settings serving homeless and non-homeless persons alike. Among these, one site (VA-A) had a component of service tailoring in that a minority of patients received primary care in shelters or a VA domiciliary, although most did not.	Team based approach / homeless-focused staff training / low threshold (walk-in) / on-site basic needs availability / integrated health care (mental/primary care) / per-workers / outreach

	Integrated health care (primary care program integrated with housing first program)	
Weinstein et al. Health Care Integration for Formerly Homeless People With Serious Mental Illness. 2013. [46]	Two multidisciplinary service teams, serving approximately 60 clients each. Partnership between an academic department of family and community medicine and the Housing First organization --> on-site primary care services added to form an integrated care program. A primary care physician, on site 2 half-days per week, within the care management team to provide primary care medical services and further linkages to primary and specialty care. Individuals may choose to receive individual psychiatric and/or primary care from the integrated care team physicians or in the community. The staff directs those with complex or acute issues to see the on-site primary care doctor if they do not already have a relationship with an outside physician. Any individual can request to see the on-site doctor. The on-site psychiatrists assess all individuals. All people in the program receive ongoing nursing assessment and care. A number of systems have been put in place to coordinate care for all individuals in the program, in addition to individual consultation between the team members about specific client concerns including a shared medical chart, monthly team medical meetings, and monthly cross-team integrated care meetings. Initiated an ongoing system of chronic disease screening and monitoring.	Multidisciplinary care, team based approach / coordinated care, integrated care (primary care/mental health/social support) / co-located services (primary care/mental health/social support) / paramedical primary care, monitoring system / care management (addressing to specialty care/further linkage to primary care)
	Universitary patient-centered medical home	
Weinstein et al. A primary care-public health partnership addressing homelessness, serious mental illness and health disparities. 2013. [47]	Partnership between an academic medical center (PCMH) and a Housing First program (pathway to Housing-PA).<br>1/ The "pathway support team" : included social workers, a nurse, a psychiatrist, a peer specialist, a vocational specialist, and a drug and alcohol counselor. All clients were supported in community-based care linkages.<br>2/ PCMH : a primary care physician from the Thomas Jefferson University Department of Family and Community Medicine was embedded into the PTH-PA Assertive Community Treatment team to provide on-site primary care and population-based health monitoring and services. Home visits were available for clients unable to come to the office. Clients could choose an outside health care provider if they wish. The staff directed individuals with acute or complex problems to the on-site physician if they have no other source of care.<br>3/ Additional clinical, evaluation, and educational partnerships have been formed with a Center for Urban Health, Department of Emergency Medicine, School of Nursing, and School of Public Health.	Multi agency partnerships (medical center/housing first program) / public health concern / multidisciplinary care / team based approach / patient-centered approach / outreach / health education of users / integrated care (primary care/mental care/housing)
	Alcohol treatment intervention based on chronic care model	
Upshur et al. A randomized control trial of a chronic intervention for homeless women with alcohol use problems. 2015. [48]	- Evidence-based training and supports to the medical leadership and randomized intervention PCPs<br>- Modifying the electronic medical record to provide alcohol screening results and alcohol-specific notes<br>- Training a Care Manager specifically designated to provide intervention participants with alcohol education materials, ongoing self-management support, linkage to formal addiction treatment services and self-help groups, wellness counseling, goal setting<br>- Intervention patients received the guideline-based primary care provider brief intervention<br>- Intervention patient's referral to the Case Manager for ongoing follow-up visits for 6 months	Collaborative care / brief intervention / specific training for primary care providers/ electronic health record / care management / self-management support / coordinated care (linkage to other services : addiction, self-group…) / well being actions (wellness counseling) / patient-centered care (goal setting)

Hewett et al. Randomised controlled trial of GP-led in-hospital management of homeless people ('Pathway'). 2016. [49]	Pathway project : nurse-led and General Practitioner (GP)-led in hospital intervention<br><br>Groupe standard care: visited once by the homelessness health nurse and provided with an information leaflet describing local services.<br><br>Groupe pathway : GP-led and nurse-led intervention involving a hospital 'in reach' team for homeless people at University College Hospital London was developed. Involved GP ward rounds and nurse practitioner patient support with a weekly multi-agency meeting. Visited regularly by the homelessness nurse to provide support and establish community links. A GP performed thrice weekly ward rounds and provided advocacy advice and medical input. A weekly multi-agency meeting was attended by the Pathway team, local council officers, hostel managers, outreach workers, drug and alcohol nurses, homeless center staff, social and palliative care workers, hospital consultants and therapists. Patients randomized to the enhanced care arm who had challenging discharge needs were discussed and multi-agency care plans devised.	Hospital "in reach" team / multi agency partnership / patient support / nurses integration / community health (linkage) / medical counseling (patient-centered care) / multi agency care plans
Lamb et al. Improving access to health care for homeless people. 2014. [50]	Streemed : nurse-led team primary care project<br><br>Nurse-led team that combines the skills of nursing and homeless outreach to bridge the gap in healthcare provision. Street Med reaches out to patients who are not accessing primary health services. Street Med meets patients in hospital, on the street, in hostels or in temporary accommodation, and carries out holistic assessments. It then takes steps to address the identified physical, mental and psychosocial health needs, taking into account the housing environment.<br>Street Med provide integrated case management by advocating across housing and health services. Street Med nurses have a sound knowledge of housing entitlement, enabling them to advocate effectively for housing.<br>Early identification of palliative care needs and improving homeless patients' access to palliative care services has been part of street Med's case management role.<br>Street Med accompanies patients to register with GP surgeries, and attends health appointments with patients, assisting with transport costs / reducing non-attendance rates.<br>Street Med also works with a homeless peer-support service called Groundswell. This service provides financial assistance and accompaniment to appointments for patients who are motivated to attend but need support to do so.	Nurses integration (nurse-led team) / outreach / whole person care / integrated care (health/social/housing) / patient support (accompaniment) / case management

O'Toole et al. Tailoring Outreach Efforts to Increase Primary Care Use Among Homeless Veterans: Results of a Randomized Controlled Trial. 2015. [36]	Outreach intervention close to primary care team<br><br>Personal health assessment/brief intervention (PHA/BI)<br>A research nurse interviewed participants and then provided feedback and a brief intervention. The outreach nurse then conducted a cursory examination. A summary of findings was then presented back to the participant explaining how information and findings may represent both untreated and undertreated medical conditions, or risks for future medical developments.<br>Clinic orientation arm (CO)<br>Immediately following assignment and receipt of the first intervention (PHA/BI or usual care), participant were transported to the clinic by the research assistant where they were introduced to the clinic team (either the patient aligned care team (PACT) or homeless PACT (H-PACT), depending on patient preference and team availability), shown where they would need to go to check-in, what the process was for being seen, as well as additional resources available at the clinic (clothes, hygiene kits, food, and benefits representatives, available to all homeless Veterans regardless of primary care enrollment), as well as where ancillary services were located. If they were unable to attend the orientation at that time, a subsequent date/time was arranged to orient them to the clinic site and staff.<br>Usual care (UC)<br>Included a social worker-administered assessment of homeless history and social needs, a description of homeless programs services, and verbal and written descriptions of clinical services, including the homeless- oriented primary care that was available at the respective Veterans Health Administration facilities, as well as the general population PACTs and clinical services, with instructions on how they could access care, where to go, and what processes and procedures were involved.	Nurses integration (research nurses) / brief intervention / patient-centered care (health assessment) / health education / multidisciplinary care / care management (orientation to services) / patient support (accompaniment) / on-site basic needs availability / integrated care (health care / Social support) / information of users
Dang et al. A web-based personal health information system for homeless youth and young adults. 2012. [51]	Health shack : web-based personal health information system<br><br>Partnership with a drop-in community agency that provided direct services and shelter to homeless youth, physicians within an academic medical center, and the software developer.<br>Characteristics of the health record : youth-centered, youth-controlled, based on internet, confidential.<br>Partnership with a pluridisciplinary team : agency staff, physicians, public health nurses, homeless youth « advocates », software developer.<br>Record included: personal health data/education/housing/working/links to comminatory resources.<br>Permitted scanning of important documents that could be securely stored online and accessed anytime by enrolled participants. Youth who enrolled in the program were also given a health identification card that listed important information such as emergency contacts, health conditions, medications, and allergies.	Electronic health medical record / multidisciplinary care / multi agency partnership (academic, drop-in community agency, shelter, medical center) / nurses integration (public health nurses) / public health concern

	Integrated primary care	
Held ML et al. Integrated primary and behavioral health care in patient-centered medical homes for jail releases with mental illness. 2012. [52]	Jail inreach project of healthcare for the homeless – Houston : Specific focus on integrated primary and mental health care for homeless individuals. Three integrated health clinics that provide comprehensive health services at no cost to individuals who are homeless. Clinics housed within existing agencies providing homeless services and shelters + essential basic needs (housing and food). Case managers provide prerelease transition planning services as well as care coordination and intensive case management after release. The case manager refers clients into needed services not provided by the project (inpatient substance abuse treatment and other systems of long-term care). Coordinated services are specific to an individual's needs and may include housing, employment, obtaining government benefits and/or identification, help with completing applications for benefits, and other services as deemed necessary. A case manager can accompany the persons to appointments with other providers. Primary care and behavioral health providers work together with the patient in the same room or with "warm" handoffs given by way of direct introduction. + Electronic health record use.	Integrated health care (mental/primary care) / multidisciplinary care / comprehensive care / low threshold (free care) / co-located services / case management / electronic health record / community health (collaboration with community agencies)
